# Time-to-infection by *Plasmodium falciparum* is largely determined by random factors

**DOI:** 10.1186/s12916-014-0252-9

**Published:** 2015-01-30

**Authors:** Mykola Pinkevych, Kiprotich Chelimo, John Vulule, James W Kazura, Ann M Moormann, Miles P Davenport

**Affiliations:** Centre for Vascular Research, University of New South Wales Australia, Kensington, Sydney NSW 2052 Australia; Kenya Medical Research Institute, Centre for Global Health Research, P. O. Box 1571, Kisumu, 40100 Kenya; Case Western Reserve University, Biomedical Research Building Suite 431, 2109 Adelbert Road, Cleveland, OH 44106 USA; University of Massachusetts Medical School, 373 Plantation Street, Room 318, Worcester, MA 01605 USA

**Keywords:** Blood-stage immunity, Malaria, Mathematical modelling, *Plasmodium falciparum*, Time-to-infection

## Abstract

**Background:**

The identification of protective immune responses to *P. falciparum* infection is an important goal for the development of a vaccine for malaria. This requires the identification of susceptible and resistant individuals, so that their immune responses may be studied. Time-to-infection studies are one method for identifying putative susceptible individuals (infected early) versus resistant individuals (infected late). However, the timing of infection is dependent on random factors, such as whether the subject was bitten by an infected mosquito, as well as individual factors, such as their level of immunity. It is important to understand how much of the observed variation in infection is simply due to chance.

**Methods:**

We analyse previously published data from a treatment-time-to-infection study of 201 individuals aged 0.5 to 78 years living in Western Kenya. We use a mathematical modelling approach to investigate the role of immunity versus random factors in determining time-to-infection in this cohort. We extend this analysis using a modelling approach to understand what factors might increase or decrease the utility of these studies for identifying susceptible and resistant individuals.

**Results:**

We find that, under most circumstances, the observed distribution of time-to-infection is consistent with this simply being a random process. We find that age, method for detection of infection (PCR versus microscopy), and underlying force of infection are all factors in determining whether time-to-infection is a useful correlate of immunity.

**Conclusions:**

Many epidemiological studies of *P. falciparum* infection assume that the observed variation in infection outcomes, such as time-to-infection or presence or absence of infection, is determined by host resistance or susceptibility. However, under most circumstances, this distribution appears largely due to the random timing of infection, particularly in children. More direct measurements, such as parasite growth rate, may be more useful than time-to-infection in segregating patients based on their level of immunity.

## Background

Infection with *Plasmodium falciparum* (*P. falciparum*) causes over 1 million deaths each year [1]. The risks of death and clinical illness is highest in young children (<5 years old), whereas adults living in endemic areas show reduced prevalence of infection, reduced parasitemia, and reduced incidence of clinical illness. This resistance to infection and illness with age is often referred to as ‘naturally acquired immunity’, and understanding the mechanisms of this may facilitate the development of a vaccine for the control of malaria. Studies of naturally acquired immunity rely on identifying variation in susceptibility in the population, and then characterizing the differences in immune responses between susceptible and resistant individuals. If immune responses associated with resistance can be identified, these may provide useful targets in the development of vaccines.

A key feature in the study of naturally acquired immunity is the identification of individuals that are relatively protected from infection or illness. If immune responses can be characterized at baseline, and subsequent infection rates identified, then it is possible to retrospectively identify those responses most closely associated with protection. Prospective cohort studies offer the opportunity to measure immune responses at baseline, and investigate these as predictors of either infection (parasitemia) or clinical illness. Susceptibility may be measured as either the presence or absence of infection or clinical episodes in a fixed time period, the number of episodes in a period, or the time to an episode. An alternative approach to studying malaria susceptibility and resistance is through a prospective study of time-to-infection in a cohort of individuals treated to eliminate malaria, and then undergoing natural exposure in an endemic setting. By observing which baseline immunological factors predict a delay in the time-to-infection, it is hoped to detect protective immune responses. Such studies have been used to explore the relationship between antibody responses and protection from both infection and clinical episodes [2-4].

Although these are generally referred to as time-to-infection studies, very different results can be obtained depending on whether infection is detected by microscopic examination of blood, or more sensitive PCR techniques [5-7]. Since these two techniques give different times of ‘infection’, it is probably more accurate to discuss these studies as measuring ‘time-to-detection’ of infection (using a particular detection method). Thus, it is important to understand that current ‘time-to-infection’ studies are always measuring ‘time-to-detection’. If we had a sensitive enough assay, the time of initiation of infection and time of detection would coincide. However, in the absence of this, we will use the term ‘time-to-initiation’ to refer to the time until initiation of blood-stage infection, and ‘time-to-detection’ to refer to what is usually described as ‘time-to-infection’.

In time-to-infection (detection) studies a major assumption is that delayed acquisition of infection (or clinical disease) is the result of the level of immune protection. However, the timing of when infection or disease is first detected depends on two major factors. The first is the random timing of when a particular individual experiences a new infection (from an infectious bite from a mosquito). The second factor is how the immune system subsequently modifies the outcome of the bite to determine whether and when infection or clinical illness is detected. For example, liver stage immunity may reduce the probability that an infectious mosquito bite results in a blood-stage infection (and only a small fraction of infected mosquito bites are thought to reach the blood stage [8,9]). Similarly, blood-stage immunity may delay the timing of parasite detection or clinical illness after the initiation of blood-stage infection, and may reduce the peak levels of parasite or the clinical manifestations of infection [5]. It is generally assumed that immunity plays a role in determining differences in time-to-infection, and thus that time-to-infection can be used as a correlate of immunity [2-4]. The major effect of pre-erythrocytic immunity would be to delay the average time-to-initiation of infection. Blood-stage immunity would not change time-to-initiation, but would change time-to-detection, because slower parasite growth would increase the delay between initiation and detection.

We have previously analysed the mechanisms of naturally acquired immunity by studying the dynamics of infection of individuals of different ages [5]. We found that the growth rate of parasites in blood-stage infection decreased with age and that this decrease in growth rate explains the differences in time-to-detection observed in individuals of different ages [5,10]. Our modelling suggested that time-to-initiation of blood-stage infection was not significantly different between age groups, and thus found little evidence for pre-erythrocytic immunity delaying time to initiation. By contrast, we found a decreased blood-stage growth with age and that this decreased growth explained the delayed time-to-detection with age. Understanding how heterogeneity in blood-stage immunity and parasite growth rate affect time-to-infection studies is important to interpreting immune correlates arising from these studies.

Herein, we have analysed the kinetics of infection in a treatment-time-to-infection (detection) study performed in Kenya [2], in order to understand the ability of this approach to identify differences in susceptibility or resistance to infection. We argue that, in most cases, the major factor that determines the time-to-detection is simply the random timing of when infection happened to be initiated. We show that, depending on the age cohort and method used to detect infection, stratifying individuals based on time-to-detection will not be useful in identifying individuals who are more susceptible or immune to infection. As a result, the timing of infection between individuals often carries little information about the level of immunity of the individuals concerned. We illustrate how the sensitivity of the method of detection of parasites can also play an important role in determining how powerful this technique is at estimating the level of immune protection; paradoxically, the higher the sensitivity of the detection method, the lower the ability to discern differences in parasite growth rate. Overall, our analysis suggests that time-to-infection studies need to be interpreted with caution, and alternative approaches such as direct measurement of parasite growth rate may be much more sensitive at detecting differences in acquired immunity to *P. falciparum* infection.

## Methods

### Field study

We analysed the data from a field study of a cohort of 201 individuals aged 0.5 to 78 years old living in a malaria holoendemic region of western Kenya [11]. This population has a high incidence of *P. falciparum* infection, which we have recently estimated as a new blood-stage infection approximately every 2 weeks [10]. Subjects were treated with Coartem®, which acts against blood-stage infection but does not affect liver-stage parasites [12]. After treatment, blood smears were monitored weekly for 11 weeks for presence of *P. falciparum* parasites by light microscopy. Individuals were removed from the study if they were found microscopy-positive by week 2 after treatment (due to presumed treatment failure) or if weekly samples were not collected after the second week of treatment, thus leaving 197 individuals for analysis. Blood samples were also later analysed using a nested polymerase chain reaction (PCR) approach to measure low levels of infection [7]. The PCR analysis was performed *post-hoc* and thus did not affect the inclusion criteria for the field study. This data was previously analysed to estimate growth rates of *P. falciparum in vivo* [5,10].

### Directly estimating the growth rate using PCR and microscopy data

We can assess the growth rate expressed as parasite multiplication rate (PMR) in individuals using the time between PCR and microscopy detection for each individual. However, we cannot estimate the growth rate precisely, since our PCR measurement shows only the presence or absence of parasites above a threshold, rather than the concentration of parasites.

**Figure 1:**
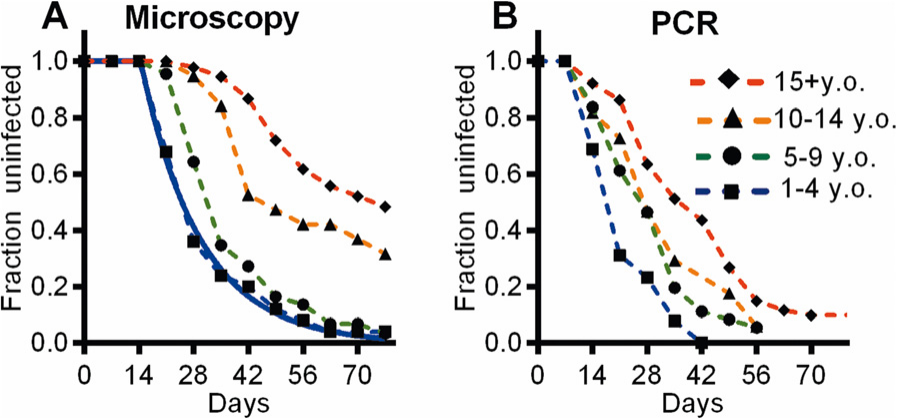
**Treatment-time-to-infection studies.** The results of a previously published treatment-time-to-infection cohort study in Kenya are shown [2]. Parasites were detected by either microscopy **(A)** or PCR **(B)**. Black shapes are data (joined by dashed lines). Black squares, blue lines – children 1 to 4 years old (y.o.); circles, green line – children 5 to 9 y.o.; triangles, orange line – children 10 to 14 y.o.; diamonds, red line – adults >14 y.o. The solid blue line is the result of fitting the exponential decay model for children 1 to 4 years old.

In order to investigate the PMR with different times-to-detection, we estimated the minimal PMR for each individual using the PCR and microscopy data (Figure 1A and B, respectively). Briefly, we identify the time of the first positive detection of parasites by PCR (*t*_PCR_) and the first detection by microscopy (*t*_micro_), and the last week when the PCR was negative (*t*_PCR_ – 7). We assume a parasite density of 40 parasites/μL as the microscopy detection threshold (*T*_*micro*_), and a density of 0.12 parasites/μL as the PCR detection threshold (*T*_*PCR*_) [5,13,14], and use the actual density of parasites at microscopy detection (*D*_*micro*_). We then estimate the parasite growth rates (PMRs), depending on the relative timing of *t*_PCR_ and *t*_micro_. If *t*_PCR_ = *t*_micro_ (i.e., parasites were first detected by PCR and microscopy on the same week), then *r =* (*D*_*micro*_*/T*_*PCR*_)^2/7^ (i.e., we assume growth from the PCR threshold to the microscopy value over the week before detection). Where *t*_PCR_ < *t*_micro_ (as was usually the case), then we know that i) parasite density was between *T*_*PCR*_ and *T*_*micro*_ at *t*_PCR_, and ii) parasite density was < *T*_*PCR*_ at (*t*_PCR_ – 7). Assuming the real parasite density was at the upper limit of these ranges (i.e., is at *T*_*micro*_ at *t*_PCR_, and at *T*_*PCR*_ at (*t*_PCR_ – 7)), we can obtain a conservative estimate of PMR, and take the larger of the two estimates. Thus,

1 $$ r= \max {\left({D}_{\mathrm{micro}}/{T}_{\mathrm{micro}}\right)}^{2/\left({t}_{\mathrm{micro}}-{t}_{\mathrm{PCR}}\right)}\Big),{\left({D}_{\mathrm{micro}}/{T}_{\mathrm{PCR}}\right)}^{2/\left({t}_{\mathrm{micro}}-{t}_{\mathrm{PCR}}-7\right)})) $$

We note, that some individuals are PCR positive, but do not become microscopy positive before the end of the study (*t*_*max*_). In this case, we assume that parasite concentration was at the microscopy detection threshold at the last week of study and estimate a maximal PMR, *r* = (*T*_*micro*_/*T*_*PCR*_)^(2/(*tmax* – *tPCR* – 7))^.

### Modelling the infection curve

In previous studies, we showed that the distribution of time to detection of infection in the different age groups are consistent with the measured reduction in PMR with age, leading to a delay in the time until the detection of infection, as well as a reduced peak of parasitaemia [5,10]. We assumed that parasites have exponential growth from the time of initiation of blood-stage infection, and a life cycle of 2 days. Thus, the concentration of parasites in blood can be described by the formula:

2 $$ C(t)=C(0){r}^{t/2} $$

Where *r* is the PMR, *C* is the concentration of parasites per μL, *t* is the time (in days) from the initiation of blood-stage infection (*t* = 0). We note that the concentration of parasites at emergence from the liver is adjusted by blood volume in each age group. In order to find the average blood volumes in age groups (*V*_*1*_ = 1.1 × 10^6^ μL, *V*_*2*_ = 2 × 10^6^ μL, *V*_*3*_ = 3.3 × 10^6^ μL, *V*_*4*_ = 5 × 10^6^ μL), we used Chart 1 in reference [15]. The number of merozoites released from the liver for a single bite we estimated as 5.6 × 10^4^ [13].

In the model, we assume that bites occur randomly with exponentially distributed times between infective bites. However, we can only detect infection after the delay θ(*r*) due to blood-stage parasite replication until the parasitemia reaches the detection threshold. This delay is equal to:

$$ \theta (r)=2 \log {}_rT/C(0) $$

where *r* is the PMR and *T* is the detection threshold (microscopy or PCR).

Assuming that the PMR is the same within age groups, we obtain the exponential decay curve until detection with initial plateau due to growth of parasites until detection.

3 $$ S(t)=\left\{\begin{array}{c}\hfill {e}^{-\lambda \left(t-2 \log {}_rT/C(0)-\tau \right)},\kern0.4em t>2 \log {}_rT/C(0)+\tau \kern0.1em \hfill \\ {}\hfill 1,\kern0.5em t\le 2 \log {}_rT/C(0)+\tau \kern6em \hfill \end{array}\kern0.1em \right. $$

The constant τ = 7 days is the first day blood-stage infection could be initiated after treatment due to the pharmacodynamics of lumefantrine [12,16-22], λ is the rate of initiation of the blood-stage infection.

We also assumed that PMR has a normal distribution within a group of people of approximately the same age with mean *m* and standard deviation *βm*, where *β* is a positive constant. This normal distribution of growth rates is consistent with the observed data, however, the precise shape of the distribution is not critical to the conclusions. Functions f(*r*) and F(*r*) are the probability density function and the cumulative density function of a normal distribution, respectively. Constant *rmax* is a maximal number of newly infected red blood cells that can be infected by one infected red blood cell.

The model that describes the infection curve with the delay to detection is defined by formula:

4 $$ \operatorname{S}(t)=\operatorname{F}(1)+1/\operatorname{F}(rmax){\displaystyle \underset{1}{\overset{rmax\kern0.1em }{\int }}{e}^{-\lambda \max \left(t-\Theta (r)-\tau, 0\right)}}\operatorname{f}(r)dr $$

This formula incorporates the initial delays to detection in exponential decay function for all possible PMRs weighted by the probability of a given PMR. The terms in front of the integral appear due to truncation of the Normal distribution at *r*_*max*_ (we assumed maximal PMR is 32 per cycle) and assumption that infections with PMR ≤1 would never be detected (function tends to plateau at F(1)).

In the current study, using assumptions of model (1), we want to find the distribution function h*(r)* of the PMR for people who were detected positive by PCR or microscopy in a given time window (*t*_1,_*t*_2_). For this purpose, we need to multiply the distribution function of the PMR f(*r*), for the whole group by the ‘fraction’ of people with given PMR in this time window, i.e., people who had initiation of blood-stage infection θ*(r)* days ago. The distribution of the PMR h(*r*) in the given time window can be found by the formula:

5 $$ \operatorname{h}(r)=k\operatorname{f}(r){\displaystyle \underset{t_1}{\overset{t_2}{\int }}{\operatorname{f}}_{\exp}\left(t-\Theta (r)\right)}dt=k\operatorname{f}(r)\left({e}^{\uplambda \max \left({t}_1-\Theta (r),0\right)}-{e}^{\uplambda \max \left({t}_2-\Theta (r),0\right)}\right) $$

The function f_exp_(*t –* Θ(*r*)) is the exponential distribution function that describes the initiation of blood-stage infection Θ(*r*) days before detection. The constant *k* normalizes the expression to make h(*r*) satisfy the condition of the probability density function (PDF).

$$ k=1/{\displaystyle \underset{1}{\overset{rmax}{\int }}\operatorname{f}(r)\left({e}^{-\uplambda \max \left({t}_1-\Theta (r),0\right)}-{e}^{-\uplambda \max \left({t}_2-\Theta (r),0\right)}\right)}dr $$

**Figure 2:**
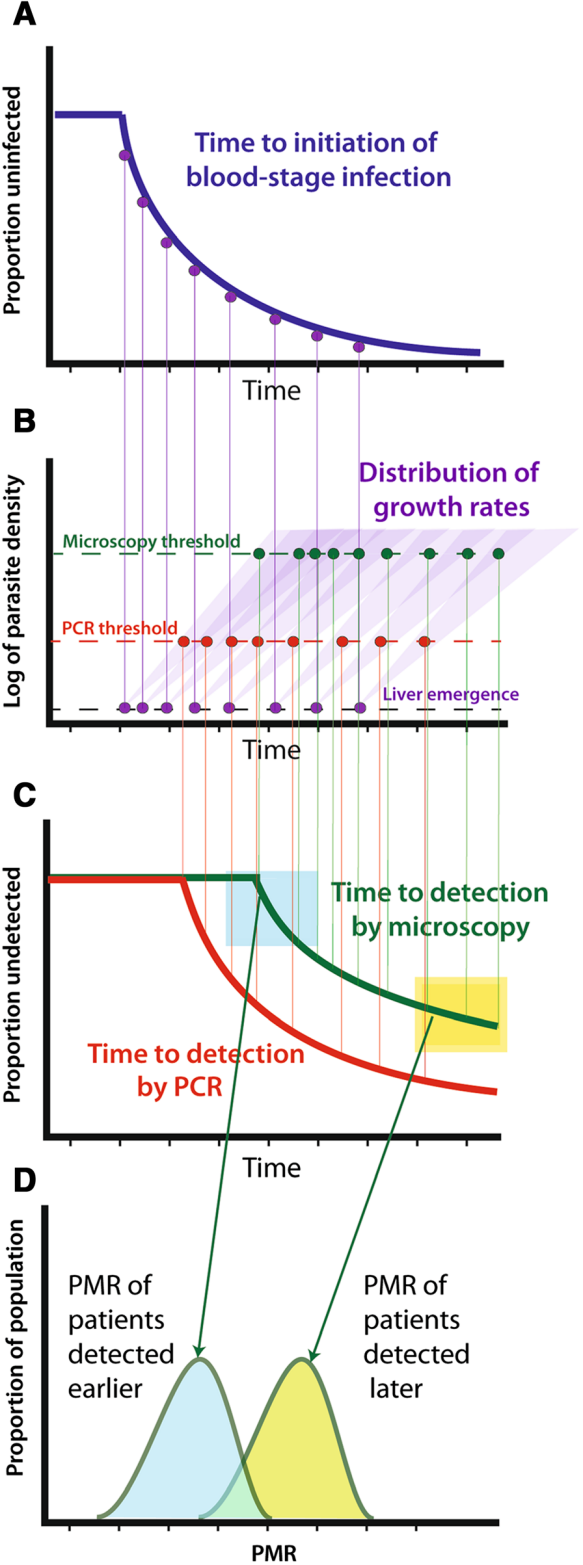
**Schematic of time-to-infection model. (A)** Given a constant force of infection, the time-to-initiation of blood-stage infection is exponentially distributed. **(B)** After emergence from the liver, there is a distribution of parasite growth rates (shaded purple triangles). **(C)** Parasites grow until they reach the threshold for detection by PCR (red dots and lines) or microscopy (green dots and lines). It is then possible to compare the growth rates for individuals detected early (blue box in **C**, blue-shaded shape in **D**) or late (yellow box in **C**, yellow-shaded shape in **D**).

The influence of PMR on delay to detection by PCR and microscopy and the difference in distributions of PMR in individuals detected earlier and later after treatment is described in the model above and is schematically illustrated in Figure 2.

## Results

### Time-to-detection in children is a random process

We first focused on the kinetics of infection of the youngest children in the cohort, aged 1 to 4 years (Figure 1A, in blue). Using detection of parasites by light microscopy, our sensitivity of detection was around 40 parasites per μL. Using this method of detection, we found that time-to-detection in these children varied from 3 weeks to 11 weeks. In order to determine whether this was a random process, we modelled time-to-detection as an exponential process using formula (3). That is, once we allow for a delay from treatment (for washout of lumefantrine, and the time taken for the parasites to grow to the level of detection), we found that the rate of detection of infection was consistent with an exponential process, with a rate of new infections (*λ*) of 0.066/day (95% CI, 0.056–0.076) (Figure 1). This equates to a ‘half-life’ (time until half the children are infected) of approximately 10 days. The exponential curve is indicative of a stochastic process, with all individuals at equal risk at all times. The close fit of the data to this model suggests that the timing of detection in these children is a random process, equivalent to radioactive decay. Thus, time-to-detection of an individual child carries essentially no information about the susceptibility of that child. The time-to-detection in these children is determined by the random time-to-initiation (of infection), and can be explained simply as a stochastic process, dependent on the time of biting.

To confirm this, we investigated whether children infected early or late differed in their subsequent infection kinetics. The blood-stage growth rate of the parasite is one factor determining when infection will be detected. Faster growing parasites should take a shorter time from emergence in the liver to reaching the detection threshold. If growth rate were the only (or major) factor determining time of detection, then early detection should be associated with faster growth, and late detection with slow growth. However, if time-to-detection is due to the random time-to-initiation of infection (as suggested above), then blood-stage growth rate will not be correlated with time-to-detection.

**Figure 3:**
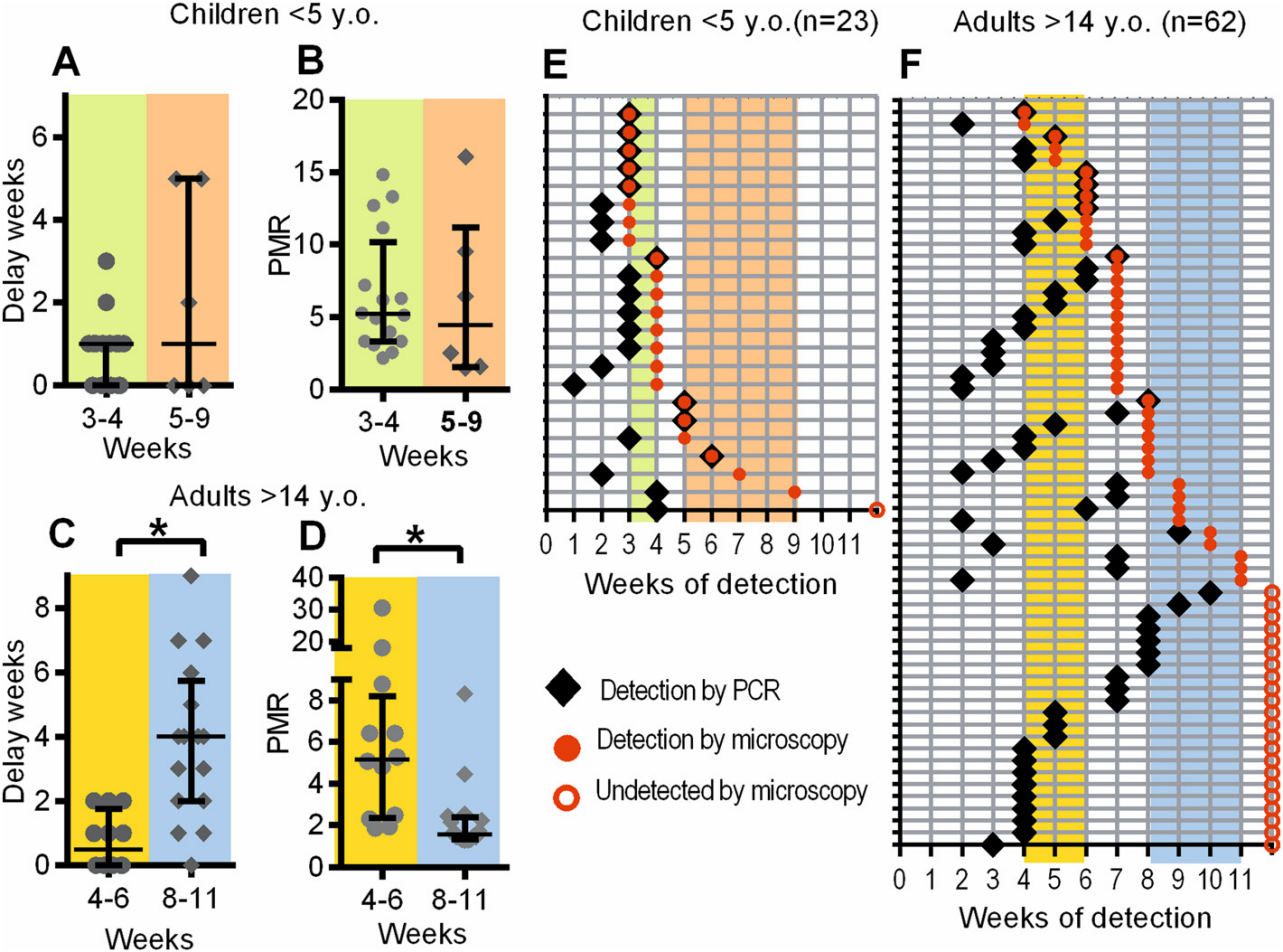
**Time-to-microscopy-detection and parasite growth rate.** Individuals were grouped according to the time at which parasites were first detected by microscopy. The time between PCR detection and microscopy detection was directly measured from the data for each patient. Parasite growth rates were estimated from the time of PCR detection to time and level of microscopy detection, using equation (1). This allowed us to compare both delays and PMR in early-detected versus late-detected groups. For children, neither the delay **(A)** nor the growth rate **(B)** was significantly different between early and late-infected groups (timing of detection shown in **E**). For adults, there was a significantly longer delay **(C)**, and slower growth rate **(D)** in the late-infected group (timing of detection shown in **F**).

One measure of blood-stage parasite growth is the time between PCR detection and microscopy detection, since this will be longer for slower growing parasites. We measured the delay between detection of parasitemia by PCR and by microscopy in children who were infected early (parasites first detected by microscopy in weeks 3 to 4 of the study) versus children infected late (weeks 5 to 9). We found no significant difference in the delay between PCR and microscopy detection in children infected early versus late (Figure 3A). We also estimated the growth rate of parasites in the same group of children (using the time of PCR detection and the level of parasitemia at microscopy detection, and equation (1)) and again found that children with different time-to-detection did not differ significantly in growth rates (Figure 3B). This suggests that blood-stage growth rate of *P. falciparum* is not a major determinant of time-to-detection in children or, conversely, that time-to-detection does not sort children based on the growth rate of blood-stage infection.

### Time to microscopy-detectable infection in adults is associated with parasite growth rate

We have previously shown that adults within this cohort had significantly different time-to-detection compared to children [5,10]. The survival curves of adults do not conform to a simple exponential reinfection process, suggesting a greater role for possible infection-modifying immune responses. Two possible mechanisms are likely to modify the time-to-detection: differences in liver-stage immunity (reducing the rate of initiation of infections), or differences in blood-stage immunity, affecting the time from liver emergence to detection of infection. We have previously demonstrated that the survival curves can be explained by heterogeneity in the growth rates of parasites in blood. If time-to-detection were caused by differences in liver-stage immunity, then we would expect no difference in parasite growth rate according to time of infection. However, if blood-stage growth rate plays a role, then we expect that adults with a longer time-to-detection would have slower parasite growth rates. Thus, we studied individuals aged >15 years in the cohort, focusing on individuals infected early (weeks 4 to 6) or late (weeks 8 to 11). Note that the designation of ‘early’ and ‘late’ infection differed considerably between children and adults, due to the different timing of detection in the different groups.

In this adult population, we find that adults with longer time-to-detection (by microscopy) have a significantly greater delay between PCR detection and microscopy detection (median 0.5 weeks versus 4 weeks, *P* value = 0.0002; Figure 3C). Using the levels of parasitemia at microscopy detection and applying formula (1), we also estimated the growth rate of parasites in adults infected early and late, and found significantly slower growth in adults infected late (median 5.156 per 2-day cycle versus 1.561, *P* = 0.0004; Figure 3D). This indicates that time-to-detection by microscopy carries information on the kinetics of blood-stage parasite growth in this adult population.

### Use of more sensitive testing reduces the ability to discriminate differences in parasite growth rate

**Figure 4:**
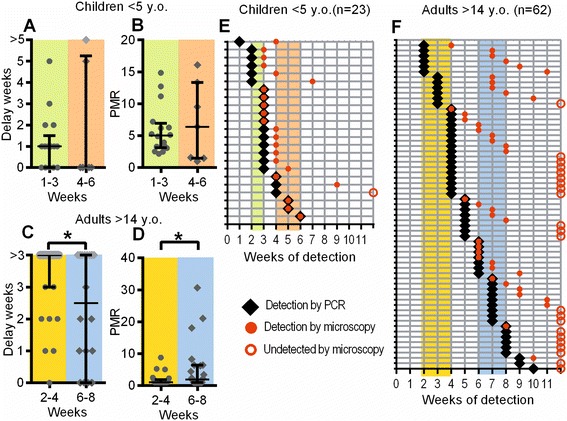
**Time-to-PCR detection and parasite growth rates.** Individuals were grouped according to the time at which parasites were first detected by PCR. The time between PCR detection and microscopy detection was measured, and PMR estimated, in order to compare parasite growth rates in early-detected versus late-detected groups. For children, neither the delay **(A)** nor the growth rate **(B)** was significantly different between early- and late-infected groups (timing of detection shown in **E**). For adults, there was a significantly shorter delay **(C)** and faster growth rate **(D)** in the late-infected group (timing of detection shown in **F**). Grey symbols in panels **A**–**D** and open circles in panels **E** and **F** indicate where parasites were not detected by microscopy before the end of the study. Note that the data in panels E and F is the same data as in Figure 2E and F, but sorted according to time-of-detection by PCR.

In addition to screening samples for infection by microscopy, we also screened samples by PCR. In the case of the children’s cohort, the shape of the reinfection curve remained exponential, despite an overall predisposition for infection to be detected earlier (Figure 1A,B, dark blue dashed lines). In the case of the adults, the shape of the time-to-detection curve is significantly altered when infection is detected by PCR (Figure 1A,B, red dashed lines). The more sensitive detection threshold of PCR causes the curve to become, overall, much more like an exponential curve. Using this data, we again stratified individuals based on time-to-detection among the children and adults, this time grouping early and late according to time of PCR detection (Figure 4). In both adults and children, there were sometimes long delays from PCR to microscopy detection. A large proportion of adults were PCR positive but were not detected by microscopy. In order not to bias the ‘late-infected cohort’ we only estimated delays/growth rates where this could be measured in both the early and late cohorts (i.e., within 3 weeks of detection by PCR in adults and 5 weeks in children).

In the children aged 1 to 5, we studied infection kinetics in individuals becoming PCR-positive in weeks 1 to 3 versus weeks 4 to 6. We observed no significant differences in either the delay between PCR positivity and microscopy positivity, or in the estimated PMR (Figure 4A,B). In adults aged >14 years we performed a similar analysis, this time comparing those who became PCR-positive in weeks 2 to 4 and in weeks 6 to 8. Here, we observed that individuals becoming PCR-positive later had a reduced time between PCR and microscopy detection (*P* = 0.0185) and an associated increased PMR (*P* = 0.0238). This is unexpected, as late-detected individuals are expected to have slower parasite growth. A confounding factor here may be the high proportion of adults who were detected as PCR-positive, but did not become microscopy-positive during the study. For these individuals we can only estimate a ‘minimum delay’ and ‘maximum growth rate’.

### Modelling time-to-detection

**Figure 5:**
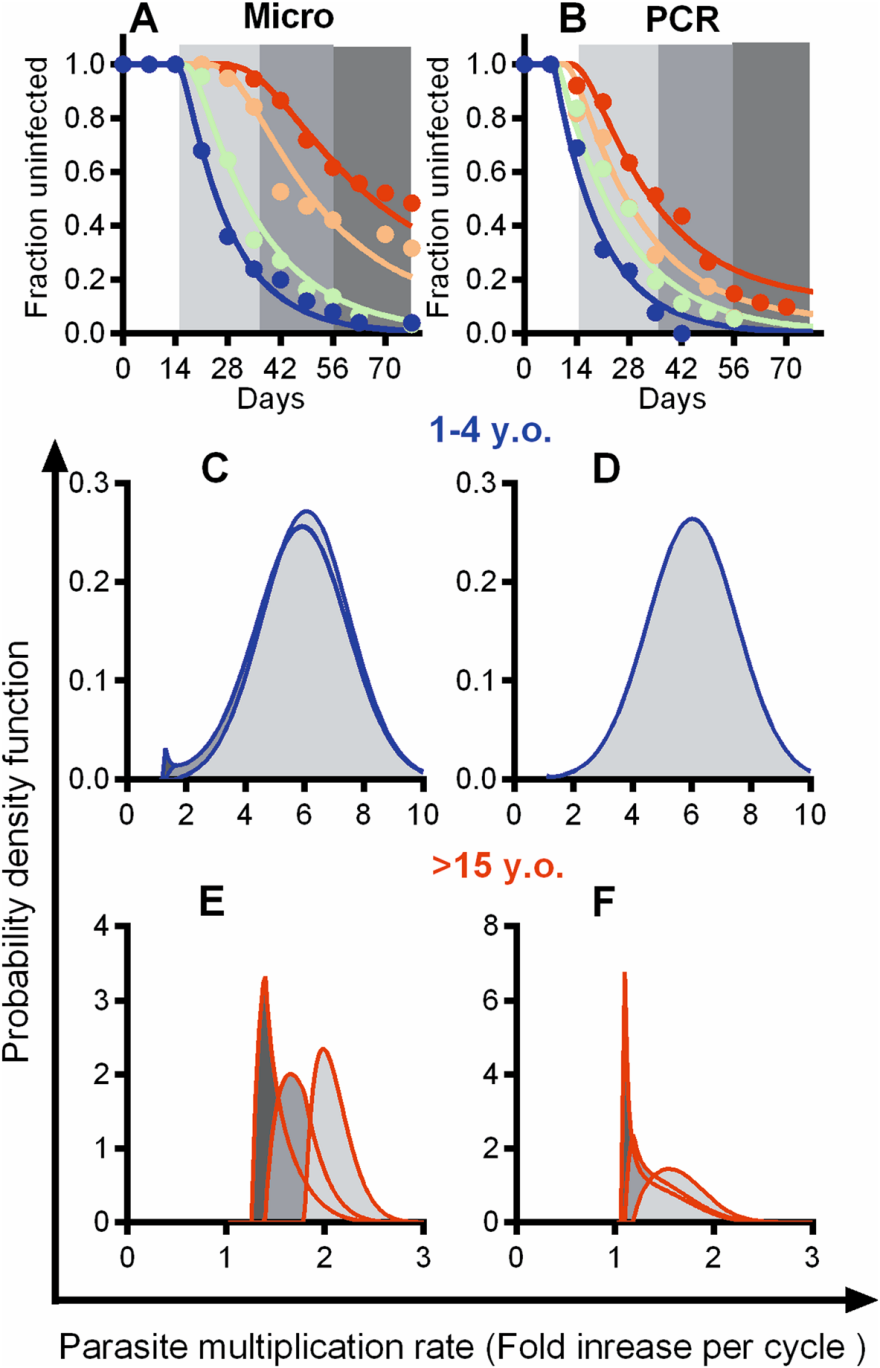
**Modelling the parasite growth rates in individuals detected at different times.** The time-to-detection of infection by microscopy **(A, C, E)** or PCR **(B, D, F)** was modelled assuming the same force of infection for all age groups. Parasite growth rates were assumed to follow a normal distribution, with a different mean parasite growth rate for children aged 1 to 5 (blue lines) and adults >14 years (red lines). Growth rate was estimated for individuals with infection detected in the first (14 to 35 days, light grey), second (35 to 56 days, medium grey), or last (56 to 77 days, dark grey) third of the study period. For children (Panels **C** and **D**), very little difference in parasite growth is predicted depending on when their infections were detected (curves for individuals detected early and late overlay each other in **C** and **D**). For adults, although the overall PMR is the same regardless of how infection is detected, microscopy is better at sorting patients based on differences in their PMR. Thus, when infection is detected by microscopy (detection threshold of 40 parasites/μL; Panel **E**), individuals infected later are predicted to have slower growth rates. When infection is detected by PCR (detection threshold of 0.12 parasites/μL; Panel **F**), there is a smaller difference in PMR between individuals infected at different times.

The analysis presented above demonstrates that the use of time-to-detection to classify individuals as susceptible or resistant to infection can be problematic in our study. This is because the random factor of when infection is initiated is often the major factor determining the time-to-detection. However, our study included a particular distribution of ages and number of individuals studied. Therefore, to illustrate the problem more generally, we used a modelling approach to look at how parasite growth rate and parasite detection method interact to determine how informative time-to-detection data is. We have previously illustrated that parasite growth is the major factor that differs between age groups in the field study. By varying only the average parasite growth rate for different age groups and assuming a normal distribution of growth rates within an age group, we found we could simultaneously fit both the PCR-determined and microscopy-determined time-to-infection curves [5,10]. These predicted differences in parasite growth rate with age were also supported by direct estimation of parasite growth rates using PCR and microscopy data for different individuals. Figure 5A,B shows the fitting of the survival curves to the microscopy and PCR detection datasets. The same model, using the predicted distribution of growth rates, was then used to understand whether time-to-detection was useful at identifying differences in parasite growth rate between ‘early infected’ and ‘late infected’ groups.

In order to illustrate the interaction of parasite growth rate and time-to-detection, we divided the youngest and oldest age cohorts (blue and red lines, respectively, in Figure 5A,B) into three groups according to time-to-detection (indicated by grey shading in Figure 5A,B). Then, we used the model to predict the expected growth rate distribution for individuals infected early (14 to 35 days), intermediate (35 to 56 days), or late (56 to 77 days) after treatment (Figure 5C–F). That is, if we model a constant force of infection and a normal distribution of growth rates within a given age group, does time-to-detection segregate individuals based on the parasite growth rates? Figure 5C shows the predicted distribution of parasite growth rates for children 1 to 4 years with infection detected by microscopy (threshold of detection = 40 parasites/μL) at different times. It is clear that, as predicted by the exponential survival curve, all groups are predicted to have very similar parasite growth rates (i.e., almost complete overlap of growth rates in Figure 5C), and the major factor determining time-to-detection is simply the random factor of when they initiated infection. By contrast, we predict that, using microscopy detection of parasites in adults, individuals detected early have a higher growth rate than individuals detected late (Figure 5E). This occurs because the much slower growth of parasites in older individuals means a much longer time between infection and detection. Thus, although the time of detection is still affected by both the random time-to-initiation (of blood-stage infection) and the subsequent growth rate, in this case, the growth rate plays a more significant role in determining time-to-detection. In our adult cohort using microscopy detection, studying early-detected and late-detected individuals allows identification of a more resistant (slower parasite growth) late-infected group.

Using the more the sensitive PCR method to detect infection (threshold of detection = 0.12 parasites/μL), the situation changes. For the children, growth rate remains overlapping for all time-to-infection groups (Figure 5D). For the adults, time-to-infection is now a much less powerful discriminator of parasite growth rate (Figure 5F show greater overlap of growth rates than Figure 5E). This occurs because the more sensitive detection reduces the time between infection and detection. Importantly, more sensitive detection reduces the delays induced by growth rate. This has more of an effect for slow growing infection (where delays are greater) than in fast growing infections. As the time separation between fast- and slow-growing infection narrows, the balance between the contribution of random infection time and differences in time from initiation to detection (due to blood-stage growth) is shifted, and time-to-detection is less affected by parasite growth rate.

### Effects of different infection rates

**Figure 6:**
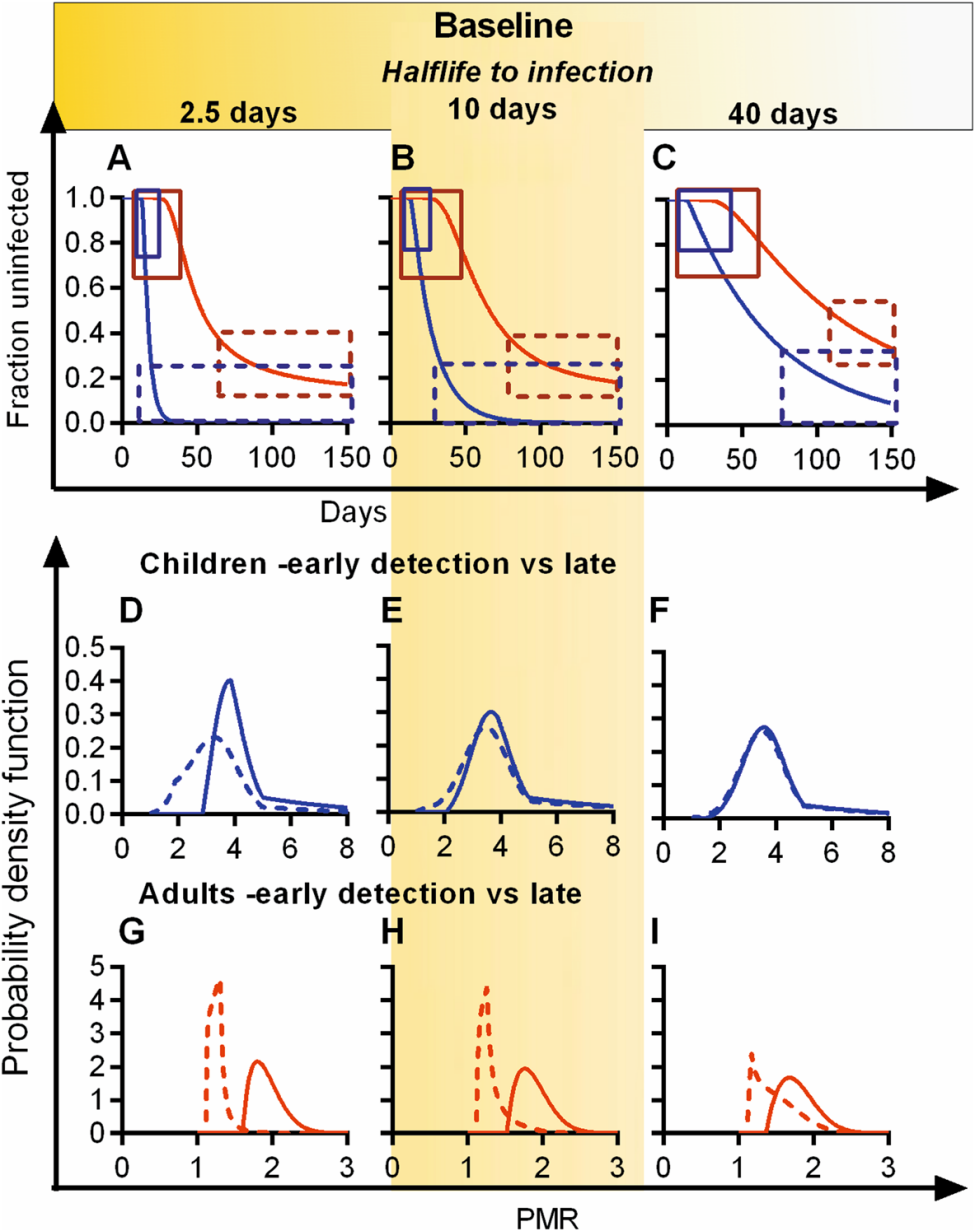
**Modelling the effects of different infection rates on time-to-infection.** Using the same model as in Figure 5, and assuming detection of infection by microscopy, we investigated the effects of raising or lowering the infection rate. The centre column (Highlighted, **B**, **E**, **H**,) shows the same infection rate as in Figure 5 (average time between bites = 10 days). Panel **A** shows the effects of a quadrupling of the infection rate. Similarly, Panel **C** shows the effects of a quartering of the infection rate. For each infection rate, the predicted time-to-infection curves for adults (red) and children (blue) are shown. Solid boxes in Panels **A**–**C** and solid lines in Panels **D**–**I** indicate an ‘early infection’ group, and dashed boxes and dashed lines indicate a ‘late infection’ group. The second row **(D–** **F)** shows the predicted difference in growth rates for children infected early versus late, at the different infection rates. Although at high infection rates **(D)** some difference in average growth rate is predicted, this is lost at lower infection rates. For adults, there is a large difference in growth rate between early- and late-infected groups at high infection rates **(G)**. However, this difference is lost at very low infection rates (Panel **I**).

The Kenyan cohort study we have analysed was performed in an area of high transmission, with an estimated entomological inoculation rate of 0.8 per day [8], and new infection rate of 0.066 per day [10]. As a result of this high infection rate, the distribution of time-to-initiation of blood-stage infections was quite short. Since the utility of time-to-detection studies in separating individuals based on growth rate of parasites is determined by the balance between delays due to infection time and delays due to subsequent growth rate, we investigated how differences in infection rate in different cohorts would affect such studies. We used the distributions in parasite growth rates for different age groups estimated from the actual cohort and then modelled the outcome for varying infection rates. Figure 6 shows how different infection rates affect the ability of time-to-infection studies to identify putatively resistant individuals with slow parasite growth. In the baseline scenario (yellow highlighted column in Figure 6), the infection rate estimated from the cohort was used. In this baseline scenario, we were able to identify differences in growth rate in adults (Figure 6H), but not in children (Figure 6E), based on time-to-infection and microscopy detection of infection (as shown also in Figure 5).

Differences in rate of initiation of infection will affect the power of the time-to-infection approach to identify more resistant or more susceptible individuals. If the biting rate had been four times higher (half-life to infection of 2.5 days; Panel A), the children would have all become infected much more quickly, meaning a shorter average delay in time-to-initiation. Therefore, even given the relatively narrow spread of parasite growth rates observed in the children, separating early- versus late-infected children would have segregated groups into higher and lower average growth rates. That is, at high infection rates, delays due to growth would have had a proportionately much larger effect on time-to-detection, and time-to-detection could be used to separate children based on parasite growth rate at this high infection rate (Figure 6D). As we lower infection rate to one quarter of the baseline rate (half-life to infection = 40 days), this reduces the differences in growth rate between early- and late-infected children (Figure 6F).

In our field study (the baseline infection rate scenario), adults infected early and late are expected to have different average growth rates (when infection is detected by microscopy) (Figure 6H). At higher infection rates (Figure 6A,G), time-to-detection remains a useful discriminator. However, when we reduced the simulated infection rate to one quarter of the baseline rate (half-life to infection of 40 days, Figure 6C), this slower infection rate significantly reduces the ability of the assay to discriminate subjects based on time-to-detection (Figure 6F,I).

The ability of time-to-infection studies to establish the more susceptible fraction of individuals (of a given age) is determined by balance of delays induced ‘waiting to be infected’ and delays in subsequent parasite growth. For the children, the delays due to differences in growth rates are small, because of the high growth rate, whereas the expected delays are much higher in adults. Since changing the infection rate does not change the PMR and the same growth rates are used for all scenarios, the time delay during growth from the liver stage to detection are independent of the infection rate. Changing the infection rate only changes the expected distribution of delays due to time-to-initiation of infection. Whenever delays due to time-to-initiation are greater than delays due to growth, time-to-detection is largely random. Only when the delays induced by growth rates are similar or higher than random delays is time-to-initiation informative. For children, this only occurs at a very high infection rate (Figure 6D). For adults, with relatively large delays due to slow parasite growth, PMR is the major determinant of delay at most infection rates shown (Figure 6G–H), but is a smaller factor at low infection rates (Figure 6I).

## Discussion

Identifying naturally acquired immune responses that are able to control parasite growth in the infected host and reduce the frequency of clinical malaria provides a potential avenue for the development of novel vaccination strategies. A number of investigators have studied how baseline (pre-treatment) immune responses affect time-to-infection after treatment [2,4]. This has also been studied using time-to-infection from cohorts that have naturally cleared malaria infection during the dry season in areas of seasonal transmission [23,24]. The underlying premise of such studies is that time-to-infection is determined by the level of immunity of the host. This assumption is supported by the fact that, when stratified by age, older individuals in endemic areas are consistently observed to have a longer time to infection, and this delay is thought to be due to naturally acquired immunity [5,25].

The association between age and time-to-infection suggests that this is a useful correlate of naturally acquired immunity. However, since the phenomenon of acquired resistance with age and exposure is well-known, comparing immune responses and time-to-infection in different age groups seems rather laborious if the same information can be obtained simply from date of birth. The major utility of time-to-infection studies would be in differentiating those of similar age and level of exposure, but who differ in their levels of immunity. By identifying the differences in immune responses between such ‘exposure matched’ individuals with different levels of protection, we should be able to identify protective responses and antigens. By comparing narrow age cohorts in localized geographical areas, we may be able to identify such responses. However, there are two major problems in this approach. First, it is also likely that such narrow cohorts may also not differ greatly in their levels of immunity, so a study design that is very sensitive to small differences in susceptibility may be required. Secondly, we are most interested in differing levels of immunity and protection in young children, as they are most at risk of clinical illness. However, since children also have the highest parasite growth rates, they are the most difficult population in which to identify differences in time-to-infection due to differences in growth rates. A major question is whether time-to-infection studies are sensitive enough to detect such differences in immunity.

Herein, we have analysed data from a time-to-infection cohort in Kenya in order to test whether such an approach is able to differentiate varying levels of protection in a group of age-matched individuals in an endemic area. We find that when infection is detected by microscopy, time-to-detection identifies adults with slower parasite growth rates; however, it does not do this well in children. When infection is detected using a more sensitive PCR approach, more adults are detected as being infected (and infection is detected earlier), but time-to-detection is less useful at identifying individuals with slower parasite growth rates. This analysis demonstrates that time-to-infection studies are very sensitive to the distribution of parasite growth rates in the group being studied, as well as the method of detection of parasites. Microscopy detection is more sensitive at segregating individuals based on parasite growth rate than PCR detection is (and using a higher threshold for detection is more sensitive still). However, in either case, the rapid growth rates of parasites in children indicate that it is very difficult to identify differences in growth rates in children using this method.

Using a simulation approach, we investigated how different rates of infection would affect the ability of time-to-detection studies to sort individuals based on parasite growth rate. This illustrates that choosing populations with higher underlying infection rates will always lead to a greater role for parasite growth rates in determining time-to-detection and thus be more sensitive at sorting individuals based on time-to-detection. Similarly, using a parasite-detection assay with a higher detection threshold will lead to an increased effect of parasite growth on time-to-detection, and thus also be more sensitive.

The mechanisms of naturally acquired immunity are generally divided into pre-erythrocytic versus blood-stage immunity. Pre-erythrocytic immunity affects the proportion of infectious bites that initiate blood-stage infection by blocking infected bites prior to or in the liver stages – thus affecting time-to-initiation (of blood-stage infection). Blood-stage immunity affects the growth rate of parasites in blood and hence the time from infection to detection. Our modelling of time-to-infection (Figure 6) reveals an inherent limitation of using such approaches to study naturally acquired immunity. Since so much of the outcome is determined by the random time-to-infection, it is very difficult to determine immune effects on parasite growth rate unless they are large. This also has potential implications for studies using time-to-infection as a means to assess vaccine effects on blood-stage parasite growth, as these may have very limited power to detect changes in time-to-detection. For studies of liver-stage vaccines the problem is slightly different, given that it is changes in the infection rate that are the primary concern (the limitations of the statistical power of such studies has been dealt with elsewhere [26]). In our previous studies [5,10], we have found no evidence for differences in infection rate with age and have shown that measured differences in parasite growth rate with age explain the observed differences in time-to-infection for different age groups. Moreover, the good fit of an exponential model of time-to-infection in children suggests little effect of pre-erythrocytic immunity. Tran et al. [23] have recently used a similar study and PCR detection to also show no difference in time-to-detection in different age groups. We note that differences in infection rate with age may make it easier to detect differences in growth rate in groups with a higher infection rate (Figure 6). It is important to understand how time-to-infection studies can be used to understand differences in parasite growth rates both in naturally acquired immunity and in studies of vaccination.

It is important to note that many studies of vaccine efficacy rely on time-to-clinical-episode, rather than time-to-infection. Infection and parasite growth are a pre-requisite for a clinical episode, and thus time-to-infection may still confound such studies. However, one approach to reducing this effect is to restrict that analysis of clinical episodes to individuals with demonstrated infection [27]. We note that, in our study, there were too few clinical episodes in the 10-week monitoring interval to allow a separate assessment of time-to-episode.

An important question is why, given the limited power of time-to-infection studies, many studies have reported significant associations between time-to-infection and both pre-erythrocytic and blood-stage immunity? One answer to this lies in the aggregation of age groups in many studies. That is, in our analysis, we considered relatively narrowly stratified age groups. If we pool all age groups, it is relatively simple to show large differences in time-to-detection with age. Since immunity also varies with age (and exposure), it is obvious that in the cohort as a whole, time-to-detection will correlate with the accumulation of immunity with age. However, since age is such a strong confounder here, it is questionable what additional information the time-to-infection adds; one could have simply correlated immune response with age and presumably reached similar conclusions. Moreover, since immunity accumulates with age and exposure, it is not possible to disentangle which immune responses are simply the result of prolonged exposure and which may be actually playing a role in reducing parasite growth rates. Where we would ideally like to identify protective responses would be in young children with similar levels of exposure but with differences in either phenotype or specificity of their immune response and who differ in infection outcome. However, in young children, we predict that time-to-infection studies are not able to discriminate differences in blood-stage immunity and parasite growth rates unless infection rates are extremely high.

Time-to-infection studies are only one approach to identify susceptible and resistant individuals. Other approaches include observing the presence or absence of infection in a given time interval, or time to presence of clinical malaria (rather than simply infection). We note that if the underlying time to acquisition of infection is a random process, then the presence or absence of infection in a given time interval is also random. For example, if we truncated our study at day 28 (Figure 1A), we would see 41% of children aged 1 to 4 infected and 59% uninfected. However, whether children were in one group or another would be due to the random distribution of time-to-initiation. Similarly, time-to-clinical-malaria is dependent on time-to-infection rate of parasite growth and underlying sensitivity to clinical malaria. Thus, the random time-to-infection may still play a dominant role. We note that others have suggested studying the rate of clinical episodes only in individuals shown to be infected [27-29]. Interestingly, this is sometimes used as a measure to decrease heterogeneity in exposure [30]. However, we suggest that this may also have the effect of reducing the impact of the random factor of when or whether infection occurred, even in the presence of homogenous levels of exposure. Further work is clearly required to determine the role of random factors versus host factors in studies of resistance to clinical malaria.

Detecting differences in parasite growth rate is difficult using time-to-infection studies, since the random timing of infection is often the major factor determining time-to-infection. Human challenge studies provide a much simpler approach for identifying differences in growth rate, as time-of-infection is known. Since all patients are infected synchronously, any delay between patients can be attributed to either a reduced initial burden of infection, or reduced subsequent growth rate. Thus, both time-to-detection and serial measurement of parasitemia can be used to estimate growth rates following infection [13,31,32]. Previous studies have shown major differences in parasite growth rates in naïve versus exposed populations [33], and similar studies could, in principle, be used to correlate prior immune responses with *in vivo* parasite growth rates following natural infection. Alternatively, the direct measurement of parasite growth rates in time-to-infection studies provides a more direct way to identify differences in blood-stage immunity than using time-to-detection in these studies. Since time-to-infection studies involve regular sampling for infection, if parasites can be detected (by PCR) in two or more sequential samples, then parasite growth rates can be directly estimated, independent of when infection was initiated. Given the significant limitations of time-to-infection studies in detecting differences in both infection rates [26] or differences in growth rates (illustrated here), we propose that direct measurement of parasite growth rates *in vivo* will be a much more useful correlate of immune control than time-to-infection itself.

## Conclusions

Many studies aim to identify resistance or susceptibility to *P. falciparum* infection based upon the timing or number of observed infections experienced by the patient. However, depending on study design, most of the variation in the timing and number of infections between age-matched individuals may arise simply from the random timing of when infection occurs. Careful attention to study design is required to identify variation in individuals’ resistance to *P. falciparum* infection.

## Abbreviations

*P. falciparum*: *Plasmodium falciparum*
PCR: Polymerase chain reaction
PMR: Parasite multiplication rate
